# Mitochondrial transplantation ameliorates experimental autoimmune encephalomyelitis by modulating the Th17/Treg balance and restoring metabolic homeostasis

**DOI:** 10.3389/fimmu.2026.1698136

**Published:** 2026-03-16

**Authors:** A Ram Lee, Suh Won Yang, Seon-Yeong Lee, Su Been Jeon, Hye yeon Kang, Jeong Won Choi, Jin Hyung Park, Ju Hyeon Park, Su Bin Son, Yunju Jeong, Jung Hwan Lee, Woojun Kim, Mi-La Cho

**Affiliations:** 1 Lab of Translational ImmunoMedicine, Catholic Research Institute of Medical Science, College of Medicine, College of Medicine, The Catholic University of Korea, Seoul, Republic of Korea; 2 Department of Pathology, College of Medicine, The Catholic University of Korea, Seoul, Republic of Korea; 3 Mitochondrial Immunomedicine center for Global Health Therapeutics (MIGHT), Catholic Research Institute of Medical Science, College of Medicine, The Catholic University of Korea, Seoul, Republic of Korea; 4 Department of Biomedicine and Health Sciences, College of Medicine, The Catholic University of Korea, Seoul, Republic of Korea; 5 Department of Food and Nutrition, College of Human Ecology, Kyung Hee University, Seoul, Republic of Korea; 6 Department of Neurology, Seoul St. Mary’s Hospital, College of Medicine, The Catholic University of Korea, Seoul, Republic of Korea

**Keywords:** experimental autoimmune encephalomyelitis (EAE), mitochondria, multiple sclerosis, spinal cord, T cell

## Abstract

**Introduction:**

Mitochondrial dysfunction has been increasingly implicated in the pathogenesis of multiple sclerosis (MS), contributing to oxidative stress, immune dysregulation, and neurodegeneration. Current therapies primarily target inflammation but do not adequately address mitochondrial impairment or progressive tissue damage. This study aimed to evaluate the therapeutic potential of mitochondrial transplantation in experimental autoimmune encephalomyelitis (EAE), a murine model of MS, by investigating its effects on immune modulation, mitochondrial function, and tissue integrity.

**Methods:**

EAE was induced in mice using myelin oligodendrocyte glycoprotein. Isolated mitochondria were administered intravenously, and clinical progression, spinal cord histology, immune cell populations, mitochondrial activity, fibrosis, and gut microbiota composition were assessed. Additionally, human peripheral blood mononuclear cells (PBMCs) from MS patients were co-cultured with mitochondria to examine ATP production, reactive oxygen species levels, and T cell differentiation.

**Results:**

Mitochondrial transplantation significantly reduced EAE severity, spinal cord inflammation, demyelination, and fibrosis. Treated mice showed increased regulatory T (Treg) cells, reduced T helper 17 (Th17) cells, improved mitochondrial biogenesis, and decreased oxidative stress. Gut microbiome analysis revealed beneficial compositional changes. In human PBMCs, mitochondrial transfer enhanced ATP synthesis, suppressed mitochondrial ROS, and promoted Treg differentiation while inhibiting pro-inflammatory cytokines.

**Discussion:**

Our findings suggest that mitochondrial transplantation restores mitochondrial function, rebalances immune responses, and mitigates neuroinflammation and fibrosis in EAE. This approach offers a novel therapeutic strategy for MS by addressing both metabolic and immunological drivers of disease progression.

## Introduction

Multiple sclerosis (MS) is a chronic autoimmune disease characterized by the immune system attacking the central nervous system (CNS), specifically targeting the myelin sheath that surrounds nerve fibers. This demyelination disrupts signal transmission, leading to neurological impairments such as sensory disturbances, motor dysfunction, and cognitive decline (1).

Current therapeutic strategies for MS primarily focus on immunomodulation; however, they show limited efficacy in preventing neurodegeneration or promoting repair, particularly in progressive forms where pathology is dominated by axonal damage and mitochondrial dysfunction (2, 3). Mitochondria are essential organelles responsible for producing ATP through oxidative phosphorylation (OXPHOS) and regulating calcium, apoptosis, and ROS generation (4, 5). While mitochondrial impairment in MS lesions contributes to energy imbalance and neuronal apoptosis (6–9). It is now increasingly recognized that mitochondrial fitness is also a fundamental determinant of immune cell fate and function. In the context of MS and EAE, the metabolic state of T cells and myeloid cells dictates their inflammatory potency. Specifically, pathogenic Th17 cells rely on aerobic glycolysis, whereas Treg cells favor mitochondrial OXPHOS to maintain immune tolerance (10). Mitochondrial dysfunction in these immune cells—characterized by impaired OXPHOS and excessive ROS—leads to the sustained activation of inflammatory pathways and a failure in immune tolerance (11–15). Additionally, the polarization of macrophages toward a pro-inflammatory M1-like phenotype is driven by a metabolic shift from OXPHOS to glycolysis, further exacerbating the inflammatory milieu (16). Therefore, mitochondrial transplantation has emerged as a promising strategy, potentially offering systemic benefits by not only providing preservation of tissue integrity but also by restoring metabolic homeostasis and reprogramming immune cell phenotypes.

Drawing upon evidence from spinal cord injury and other CNS injury models—where transplanted mitochondria have been shown to be actively internalized by host cells such as macrophages and axons to restore bioenergetic function (17, 18) exogenous mitochondrial delivery represents a novel approach to metabolically “rescue” compromised cells.

Mechanistically, it has been established that the intercellular transport of mitochondria is a regulated process, often facilitated by Miro1-mediated movement through tunneling nanotubes, which enhances the rescue efficacy in stressed environments (19). Furthermore, studies have demonstrated that these extracellular mitochondria can successfully transit into host cells and integrate into the endogenous mitochondrial network to maintain functional integrity (20).

Preclinical studies have demonstrated that introducing healthy mitochondria into damaged neural tissues can restore mitochondrial function, reduce inflammation, and improve cognitive and motor functions, in models of traumatic brain injury and ischemia (18, 21), Parkinson’s and Alzheimer’s diseases (22, 23), and status epilepticus (24). However, despite these advancements in local CNS injury models, the systemic impact of mitochondrial transplantation on the overall immune landscape of the EAE model remains unexplored.

This study aims to explore the impact of mitochondrial transplantation in an EAE mouse model of MS, focusing on how restoring mitochondrial function through transplantation could modulate immune responses and inflammation, potentially altering disease outcomes. We examined how mitochondrial transplantation influences immune regulation and pathological changes in EAE. Specifically, we analyzed the proportions of key T-cell subsets and evaluated fibrotic markers in spinal cord tissues to elucidate the immunomodulatory and anti-fibrotic effects of mitochondrial therapy.

## Materials and methods

### Animals

Seven-week-old male C57BL/6 mice were purchased from Orient Bio (Seongnam, Korea). All animals were maintained under specific-pathogen-free (SPF) conditions with ad libitum access to water and a standard laboratory diet (Ralston Purina, St. Louis, MO, USA). All experimental procedures were approved by the Institutional Animal Care and Use Committee of the Catholic University of Korea (ID number: CUMC-2020-0048-02) and were performed in accordance with the ethical guidelines for animal experimentation.

### Induction of experimental autoimmune encephalomyelitis

Mice were immunized via subcutaneous injection with 500 μg of MOG35–55 peptide emulsified in incomplete Freund’s adjuvant (Chondrex, Redmond, WA, USA) supplemented with 500 μg of inactivated *Mycobacterium tuberculosis* (Difco, Franklin Lakes, NJ, USA). The emulsion was administered at two separate sites on the bilateral flanks. Additionally, 200 ng of pertussis toxin (Sigma, St. Louis, MO, USA) was administered Intraperitoneal Injection on days 0 and 2 to facilitate the induction of the disease. The mice were then observed and scored on a scale of 0-5 (with gradations at 0.5 intervals, allowing for intermediate scores): 0, no clinical signs; 1, loss of tail tone; 2, wobbly gait; 3, hindlimb paralysis; 4, hindlimb and forelimb paralysis; and 5, death. The pathology scoring was conducted by two proficient technicians using a blind test. Each group contained five mice, and all experiments were repeated three or more times. All animals were euthanized using carbon dioxide (CO_2_) inhalation in accordance with the AVMA Guidelines for the Euthanasia of Animals. CO_2_ gas was introduced into the chamber at a controlled rate of 40% of the chamber volume per minute, following the displacement method recommended by the guidelines. Pre-filling of the chamber was avoided. Animals were monitored for complete cessation of respiration and heartbeat to confirm death.

### Isolation of mitochondria from L6 cells

Mitochondria were isolated from L6 rat skeletal muscle cells using a Mitochondrial Isolation Kit for Cultured Cells (89874; Thermo, Waltham, MA, USA). In summary, 3×10^6^ L6 cells were incubated with 400 μL of reagent A for 2 min on ice, with vortexing occurring every minute, and subsequently incubated with 5 μL of reagent B for 5 min on ice. Following the addition of 400 μL of reagent C, the cells were subjected to a centrifugation process at 700 × g for a duration of 10 min at a temperature of 4 °C. The resultant pellet was then transferred to a new tube and subjected to a further centrifugation at 12,000 × g for 15 min at 4 °C. The resulting pellet (mitochondria) was then washed with 400 μL of reagent C and subsequently subjected to a final centrifugation at 12,000 × g for 5 min at 4 °C. Protein concentrations were then quantified by bicinchoninic acid assay (23235; Thermo). In the EAE mouse model, mitochondria were administered intravenously via the tail vein once weekly at a dose of 10 μg in a total volume of 1 mL, whereas control mice received an equivalent volume of saline.

### Splenocyte isolation and culture

Splenocytes were isolated from the spleens of the mice. The spleens were harvested and dissociated to obtain a single-cell suspension. To remove red blood cells, the cells were treated with ACK (Ammonium-Chloride-Potassium) lysing buffer, followed by centrifugation at 1,500 rpm for 5 minutes at 4 °C. The isolated splenocytes were re-suspended and cultured in RPMI 1640 medium supplemented with 5% bovine serum albumin (BSA).

### Isolation of human PBMCs

The study protocol was approved by the Institutional Review Board (IRB) of Seoul St. Mary’s Hospital, The Catholic University of Korea (IRB number: KC24TISI0369), and written informed consent was obtained from all participants. All procedures were performed in accordance with the ethical standards of the Declaration of Helsinki. PBMCs were obtained from three female patients diagnosed with relapsing-remitting multiple sclerosis (RRMS). The patients were 20, 30, and 31 years old, with disease durations of 2.22, 7.19, and 12.52 years, respectively. All patients were receiving treatment with interferon beta-1a and were in a remission phase at the time of blood sampling. PBMCs were isolated from the peripheral blood of healthy controls (HCs) and patients with MS using Ficoll-Hypaque density gradient centrifugation (density 1.077 g/mL; Pharmacia Biotech, Uppsala, Sweden). Diluted blood was layered on Ficoll and centrifuged at 400 × g for 30 minutes (brake off). The mononuclear cell layer was harvested, washed twice with PBS, and treated with RBC lysis buffer to ensure purity. The isolated PBMCs were then resuspended in RPMI 1640 medium supplemented with 10% fetal bovine serum (FBS) and 1% penicillin-streptomycin for further experiments.

### Histopathological analysis

After euthanasia, mice were transcardially perfused with cold PBS and fixed with 10% neutral buffered formalin. The spinal cords were then dissected and processed for paraffin embedding. Five-micrometer-thick sections were prepared using a microtome (Leica Biosystems, Wetzlar, Germany). To evaluate pathological changes, sections were stained with Hematoxylin and Eosin (H&E) to assess inflammatory cell infiltration, and Luxol Fast Blue (LFB) with a hematoxylin counterstain to visualize the extent of demyelination. Pathological lesions were quantified using a semi-quantitative scoring system on a scale of 0 to 4. For inflammatory infiltration (H&E), the scores were defined as: 0, no infiltration; 1, small infiltration; 2, < 1/3 of white matter infiltrated; 3, > 1/3 of white matter infiltrated; and 4, severe inflammation. For demyelination (LFB), the scores were: 0, no demyelination; 1, mild demyelination; 2, < 1/3 of the demyelination area; 3, > 1/3 of the demyelination area; and 4, severe demyelination. All evaluations were performed by two independent observers in a blinded manner.

### Flow cytometry and cell identification

To confirm the delivery of exogenous mitochondria and evaluate immune cell subsets, flow cytometry was performed using a FACS Calibur instrument (BD Biosciences, San Jose, CA, USA). To track mitochondrial transfer, L6 cells were pre-labeled with 200 nM MitoTracker Deep Red (MTDR; M22426, Thermo Fisher Scientific, Waltham, MA, USA) for 30 minutes at 37 °C before mitochondrial isolation, ensuring that only the exogenous mitochondria were tracked upon delivery to recipient cells. These pre-labeled mitochondria were then cocultured with human PBMCs to confirm their successful internalization into the recipient cells. To assess mitochondrial ROS levels, treated human PBMCs were incubated with 5 μM MitoSOX (M36008; Thermo Fisher Scientific) for 10 minutes at 37 °C. For the identification of T cell subsets, harvested cells were stained with species-specific fluorochrome-conjugated antibodies at a 1:100 dilution. For intracellular markers, including IL-17A and Foxp3, cells were fixed and permeabilized using the Foxp3/Transcription Factor Staining Buffer Kit (eBioscience Waltham, MA, USA) according to the manufacturer’s instructions. To identify human Th17 cells, PBMCs were stained with PE-Cy7-conjugated anti-CD4 (BioLegend, 300512) and PE-conjugated anti-IL-17A (eBioscience, 12-7179-42), while human Treg cells were identified using PE-Cy7-conjugated anti-CD4, APC-conjugated anti-CD25 (BioLegend, 302610), and FITC-conjugated anti-Foxp3 (eBioscience, 11-4776-42). Similarly, to identify mouse Th17 cells, splenocytes were stained with PerCP-Cy5.5-conjugated anti-CD4 (eBioscience, 45-0042-82) and APC-conjugated anti-IL-17A (eBioscience, 17-7177-81), whereas mouse Treg cells were identified using PerCP-Cy5.5-conjugated anti-CD4, APC-conjugated anti-CD25 (BioLegend, 102012), and PE-conjugated anti-Foxp3 (eBioscience, 12-5773-82). All flow cytometric data were analyzed using FlowJo software (TreeStar, Ashland, OR, USA).

### Enzyme-linked immunosorbent assay

Serum levels of MOG-specific IgG2 were determined by indirect ELISA. 96-well plates were coated overnight at 4 °C with 10 μg/mL of MOG35–55 peptide in carbonate buffer. After blocking with 1% BSA in PBS for 1 hour, diluted serum samples were added to the wells and incubated for 2 hours at room temperature. The levels of MOG-specific antibodies were then detected using the Mouse IgG2 ELISA Quantitation Set (A90-107P, Bethyl Laboratories, Montgomery, TX, USA) according to the manufacturer’s instructions. The absorbance was measured at 405 nm using an ELISA microplate reader (Molecular Devices, San Jose, CA, USA).

### Immunohistochemistry

For immunohistochemical analysis, paraffin-embedded spinal cord sections were deparaffinized and rehydrated. The sections were then incubated overnight at 4 °C with the following primary antibodies at a 1:200 dilution: anti-TOM20 (ab56783; abcam), anti-TFAM (22586-1-AP; Proteintech), anti-PINK1 (BC100-494; Novus Biological), anti-IL-17 (ab79056; abcam), and anti-TNFα (ab6671; abcam), anti-iNOS (PA3-030A; Invitrogen), anti-COL1A1 (PA5-29569; Invitrogen), and anti-α-SMA (ab7817; abcam), Sections were incubated with secondary biotinylated antibodies, then incubated with streptavidin–peroxidase complex for 30 min. Reaction products were developed using 3,3-diaminobenzidine chromogen (K3468; Dako Corp.).

### Confocal microscopy

Paraffin-embedded spinal cord sections stained with phycoerythrin (PE)-conjugated rat anti-mouse CD4 (45-0042-82; eBioscience), fluorescein isothiocyanate (FITC)-conjugated rat anti-mouse IL-17A (11-7177-81; eBioscience), and FITC-conjugated rat anti-mouse CD25 (102006; BioLegend) and rat allophycocyanin (APC)-conjugated mouse anti-Foxp3 (77-5775-40; eBioscience) Antibodies were applied to the samples and left to incubate at 4 °C for 16 hours. The stained samples were then observed under a Zeiss confocal microscope (LSM 510 Meta; Carl Zeiss, Jena, Germany). The enumeration of stained cells was conducted by two proficient technicians by a blind test. Each group contained five mice, and all experiments were repeated three times.

### ATP assays

To evaluate the functional integration and metabolic impact of mitochondrial transfer, mitochondria isolated from the L6 cell pre-labeled with MTDR as described above—were cocultured with human PBMCs. ATP production was conducted utilizing an ATPlite luminescence assay system (PerkinElmer, 6016943). Following the confirmation of intracellular internalization, 5 x 10^5^ human PBMC/well were seeded into black 96-well plates. To measure ATP synthesis, the cells were incubated with 2 mM ADP for 45 min at 37 °C. Subsequent to this, 50 μL of mammalian cell lysis solution was added, and the plate was shaken for five minutes in an orbital shaker at 700 rpm. After 5 min, 50 μL of substrate solution were added, and the plate was shaken under the same conditions. Luminescence was subsequently measured using a SpectraMax L (Molecular Devices Inc., Sunnyvale, CA, USA).

### Analysis of gut microbiome

Fecal samples were collected from vehicle-treated and mitochondrial transplantation groups at the time of sacrifice (Day 22 post-induction). Following the procedure described previously (Gut Microbes, 2024, 16:1, 2300846), samples were stored at −70 °C within 12 hours of collection. Total genomic DNA was extracted using the FastDNA^®^ SPIN Kit for Soil (MP Biomedicals, Solon, OH, USA). For microbial profiling, the V3–V4 regions of the 16S rRNA gene were amplified using fusion primers 341F (5′-CCTACGGGNGGCWGCAG-3′) and 805R (5′-GACTACHVGGGTATCTAATCC-3′). PCR was performed under the following conditions: initial denaturation at 95 °C for 3min, followed by 25 cycles of denaturation at 95 °C for 30 s, annealing at 55 °C for 30 s, extension at 72 °C for 30 s, and a final elongation at 72 °C for 5 min. Sequencing was performed by Macrogen Inc. (Seoul, Korea) using the Illumina MiSeq platform. Raw sequences were processed and analyzed using the QIIME 2 pipeline (version 2019.4) to determine the gut microbiome composition.

### Statistical analysis

Results are presented as means ± standard errors of the mean. The normality of the data distribution was assessed using the Shapiro-Wilk test. Data were analyzed by Student’s t-test or the Mann–Whitney U test using Prism 5 software (GraphPad Inc., San Diego, CA, USA). P-values < 0.05 (two-tailed) were considered indicative of statistical significance.

## Results

### Mitochondrial transplantation attenuates disease progression and spinal cord pathology in EAE model

**Figure 1:**
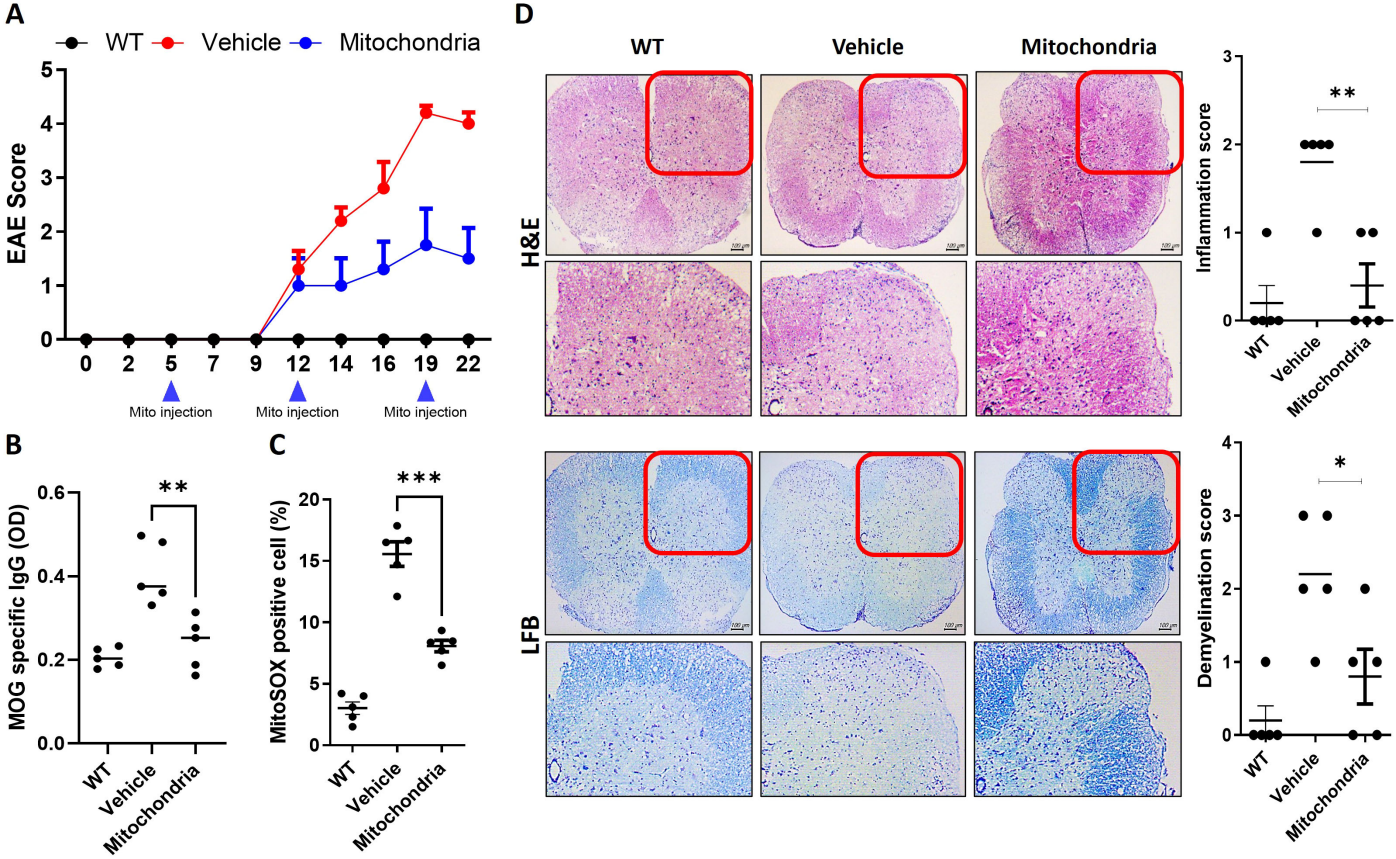
Clinical and histological findings in experimental autoimmune encephalomyelitis (EAE) after mitochondrial transplantation. **(A)** Clinical scores assessed in saline-injected (vehicle) and mitochondria-injected mice throughout the course of EAE. Data are shown as representative results (mean ± SEM) from three independent experiments (n=5 per group) **(B)** Concentrations of MOG-specific IgG in serum quantified by enzyme-linked immunosorbent assay. **(C)** Mitochondrial ROS levels measured by MitoSOX fluorescence analyzed in splenocyte populations using *ex vivo* flow cytometry. **(D)** Spinal cord sections subjected to H&E (hematoxylin and eosin) staining to assess inflammatory cell infiltration and LFB (Luxol Fast Blue) staining to evaluate demyelination666. Enlarged insets (magnified views) are provided to highlight perivascular infiltration and myelin loss. *P < 0.05, **P < 0.01, ***P < 0.001.

To evaluate the therapeutic efficacy of mitochondrial transplantation in the EAE model, we first monitored the clinical progression of the disease. While healthy naïve mice typically maintain a score of 0, the vehicle-treated EAE group showed a rapid increase in disease severity starting from day 12 post-induction, reaching a peak score of approximately 4.0. In contrast, mice administered mitochondria intravenously exhibited a significantly lower clinical score throughout the observation period, with the peak score suppressed to below 2.0 (**Figure 1A**). This suggests that systemic mitochondrial delivery effectively delays the onset and reduces the overall severity of EAE. To investigate the impact on the autoimmune response and oxidative stress, we measured MOG-specific IgG levels and mitochondrial ROS (mtROS) production. Mitochondrial transplantation resulted in a significant reduction of MOG-specific IgG in the serum (**Figure 1B**), indicating a suppression of the adaptive autoimmune response against myelin. Furthermore, the percentage of MitoSOX-positive cells an indicator of mitochondrial superoxide production and oxidative stress in immune cells was markedly decreased in the mitochondria-treated group compared to the vehicle group (**Figure 1C**). The protective effect on spinal cord structural integrity was further validated through histological analysis of the spinal cord tissues. We employed H&E staining to visualize inflammatory cell infiltration and LFB staining to assess the extent of white matter demyelination. In the vehicle-treated group, the enlarged insets clearly demonstrate extensive perivascular and parenchymal infiltration of inflammatory cells, characterized by marked hyper-cellularity within the white matter. was observed, coinciding with focal areas of demyelination within the white matter (**Figure 1D**). However, the mitochondria-treated group showed a marked reduction in both inflammatory infiltration and myelin damage. Quantitative scoring confirmed these findings, with significantly lower inflammation and demyelination scores in the treated group compared to controls. When compared to the baseline integrity expected in naïve mice, mitochondrial transplantation significantly preserved the structural architecture of the spinal cord, demonstrating its potent anti-inflammatory and myelin-protective effects.

### Mitochondrial transplantation promotes regulatory T cell differentiation and suppresses Th17 responses in EAE model

**Figure 2:**
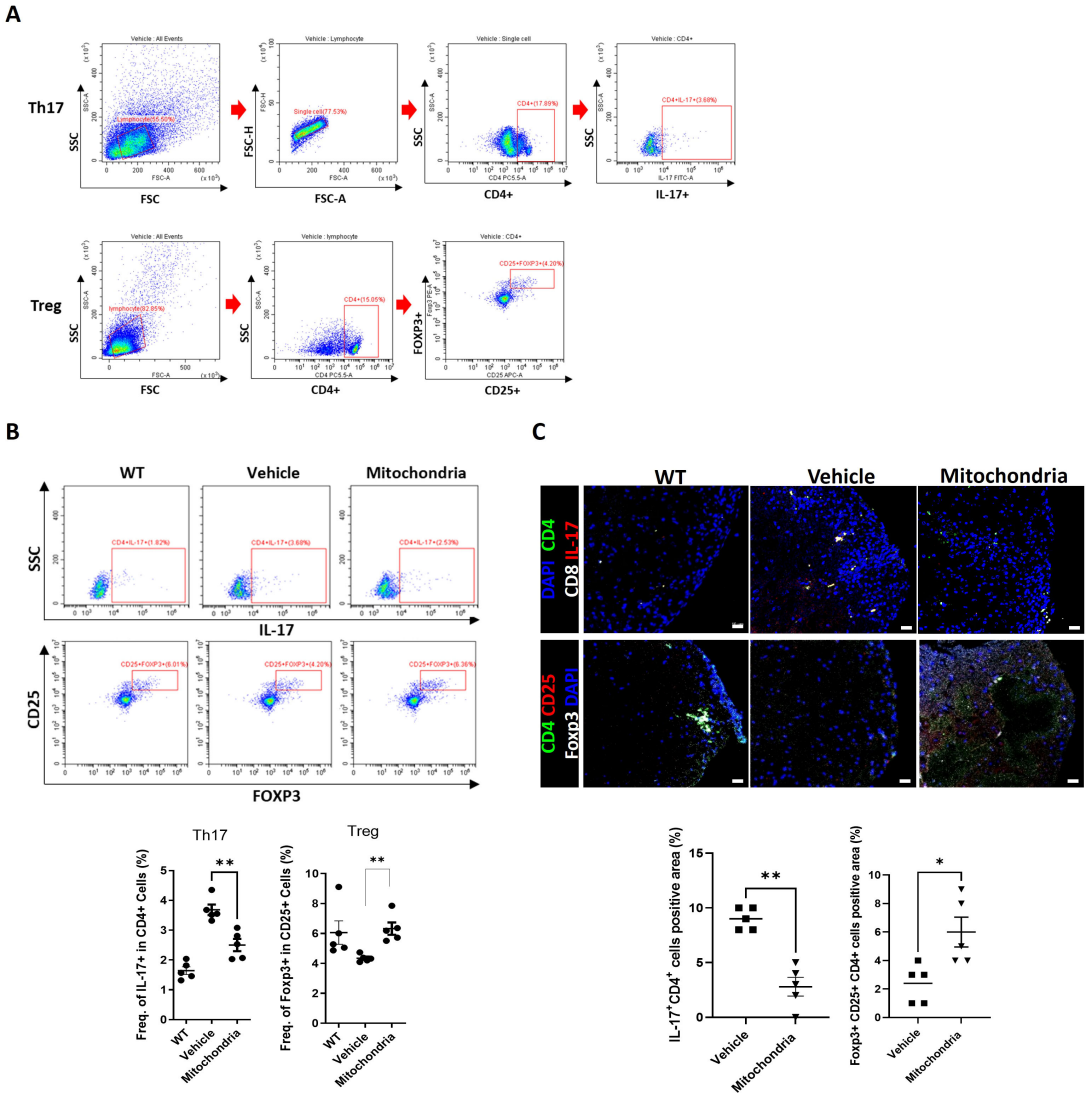
Regulatory T cell differentiation after mitochondrial transplantation in splenocytes and spinal cord tissues in an EAE model harvested 22 days after induction. **(A)** Representative gating strategy for the flow-cytometric identification of CD4+ T cell subsets. **(B)** The frequencies of T helper 17 (Th17) and regulatory T (Treg) cells within the splenocyte populations quantified using *ex vivo* flow cytometry. **(C)** Spinal cord sections analyzed by confocal microscopy. Scale bars = 20 μm. *P < 0.05, **P < 0.01.

To investigate whether the clinical improvement was associated with the modulation of systemic and local adaptive immune responses, we analyzed T cell subsets in both the peripheral lymphoid organ and the CNS. Given that EAE is characterized by an imbalance between pro-inflammatory Th17 cells and anti-inflammatory Tregs, we hypothesized that mitochondrial transplantation would promote a more tolerogenic immune profile. First, we examined the systemic immune response using immune cells isolated from the splenocytes of EAE mice via flow cytometry (**Figures 2A, B**). In the vehicle-treated EAE group, a high frequency of Th17 cells (CD4^+^IL-17^+^) was observed, representing a pathological expansion compared to the low levels typically found in naïve mice. However, the mitochondrial transplantation group showed a significant decrease in the frequency of IL-17^+^ cells within the CD4^+^ population. Conversely, the frequency of Tregs (CD4^+^CD25^+^FOXP3^+^) was markedly increased in the mitochondria-treated group compared to the vehicle control. This shift in the Th17/Treg ratio in the spleen suggests that systemic mitochondrial delivery recalibrates the adaptive immune system towards a regulatory phenotype. Next, we evaluated whether these immune-modulatory effects extended to the site of CNS injury by examining T cell activity in the spinal cord tissue using immunofluorescence staining (**Figure 2C**). Consistent with the systemic findings, the infiltration of Th17 cells into the spinal cord lesions was significantly reduced in the mitochondria-treated group compared to the vehicle group. In contrast, the number of infiltrating Tregs was significantly higher in the treated group. Our results indicate that mitochondrial transplantation effectively limits the presence of encephalitogenic Th17 cells while facilitating the recruitment or expansion of protective Tregs at the disease site.

### Mitochondrial transplantation enhances mitochondrial activity in spinal cord

**Figure 3:**
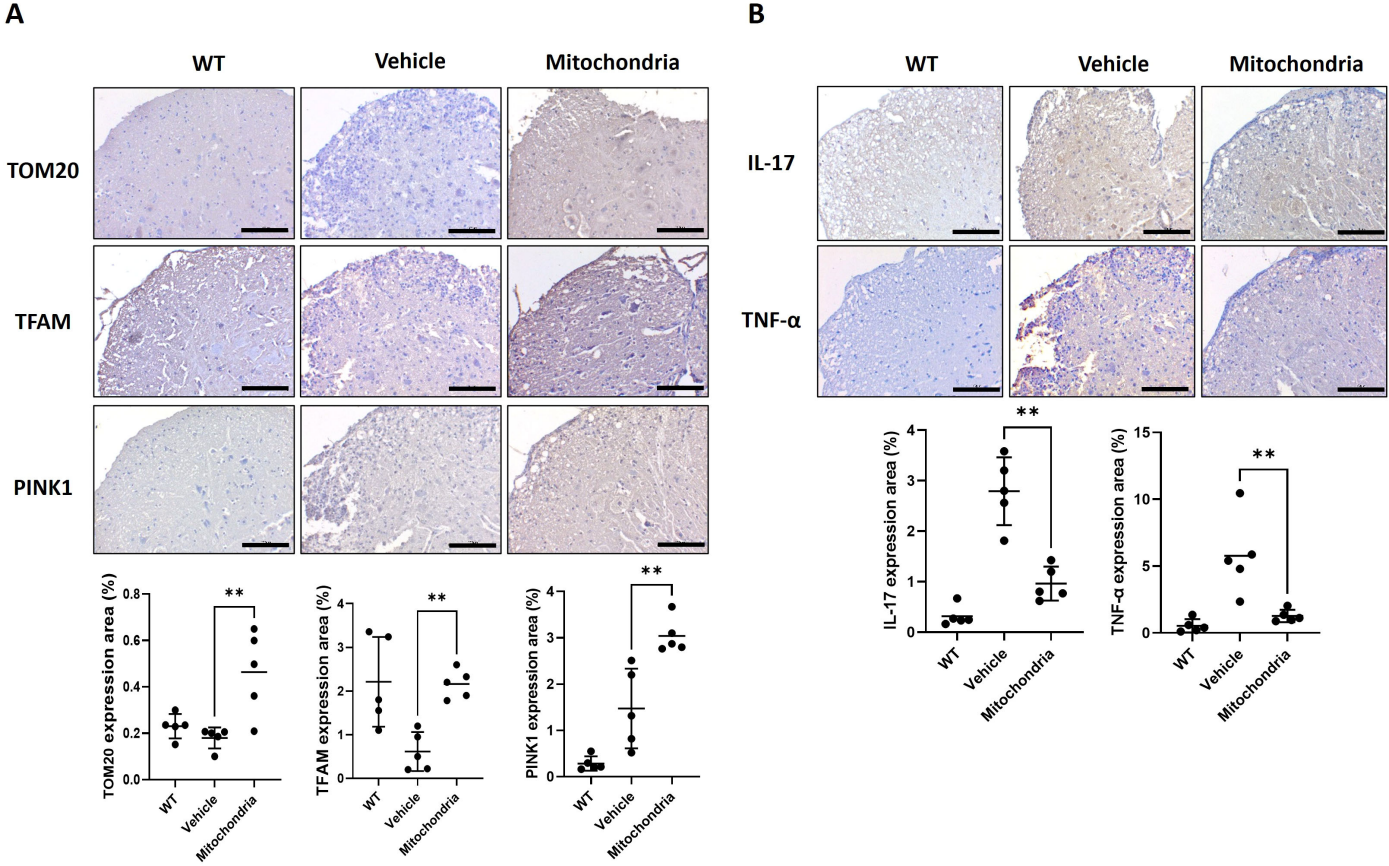
Mitochondrial markers after mitochondrial transplantation in spinal cord tissue of an EAE model. **(A)** Expression levels of mitochondrial biogenesis markers TOM20, TFAM, and PINK1 in spinal cord tissues evaluated by immunohistochemistry. Scale bars = 100 μm. **(B)** Expression levels of inflammatory cytokines IL-17 and TNF-α in spinal cord tissues evaluated by immunohistochemistry. Scale bars = 100 μm. **P < 0.01.

To investigate whether the observed therapeutic effects were associated with the restoration of host mitochondrial health and quality control within the CNS, we analyzed the expression of key mitochondrial biogenesis and stability markers in spinal cord tissues. We assessed TOM20, a protein of the mitochondrial outer membrane; TFAM, a key activator of mitochondrial DNA transcription; and PINK1, a master regulator of mitophagy and mitochondrial quality control, to comprehensively assess mitochondrial content and maintenance. Immunohistochemical (IHC) analysis revealed that mitochondrial transplantation significantly increased the positive expression areas of TOM20, TFAM, and PINK1 in the spinal cord lesions compared to the vehicle-treated EAE group (**Figure 3A**). The significant upregulation of TFAM and PINK1, that the treatment not only provides exogenous mitochondrial support but also reactivates the endogenous mitochondrial biogenesis and quality control systems of the host tissue. Furthermore, we examined whether this restoration of mitochondrial homeostasis was accompanied by a reduction in localized neuroinflammation. We measured the expression of IL-17 and TNF-α, which are hallmark pro-inflammatory cytokines in the pathogenesis of EAE. In the vehicle-treated group, high levels of IL-17 and TNF-α were observed throughout the spinal cord parenchyma, reflecting an active inflammatory state (**Figure 3B**). In contrast, the mitochondrial transplantation group exhibited a significant reduction in the expression of both cytokines. This attenuation of neuroinflammatory markers correlates with the recovered mitochondrial integrity, suggesting that mitochondrial therapy mitigates disease progression by suppressing the local inflammatory milieu and fostering a more permissive environment for tissue stability.

### Mitochondrial transplantation inhibits fibrosis marker in spinal cord injury

**Figure 4:**
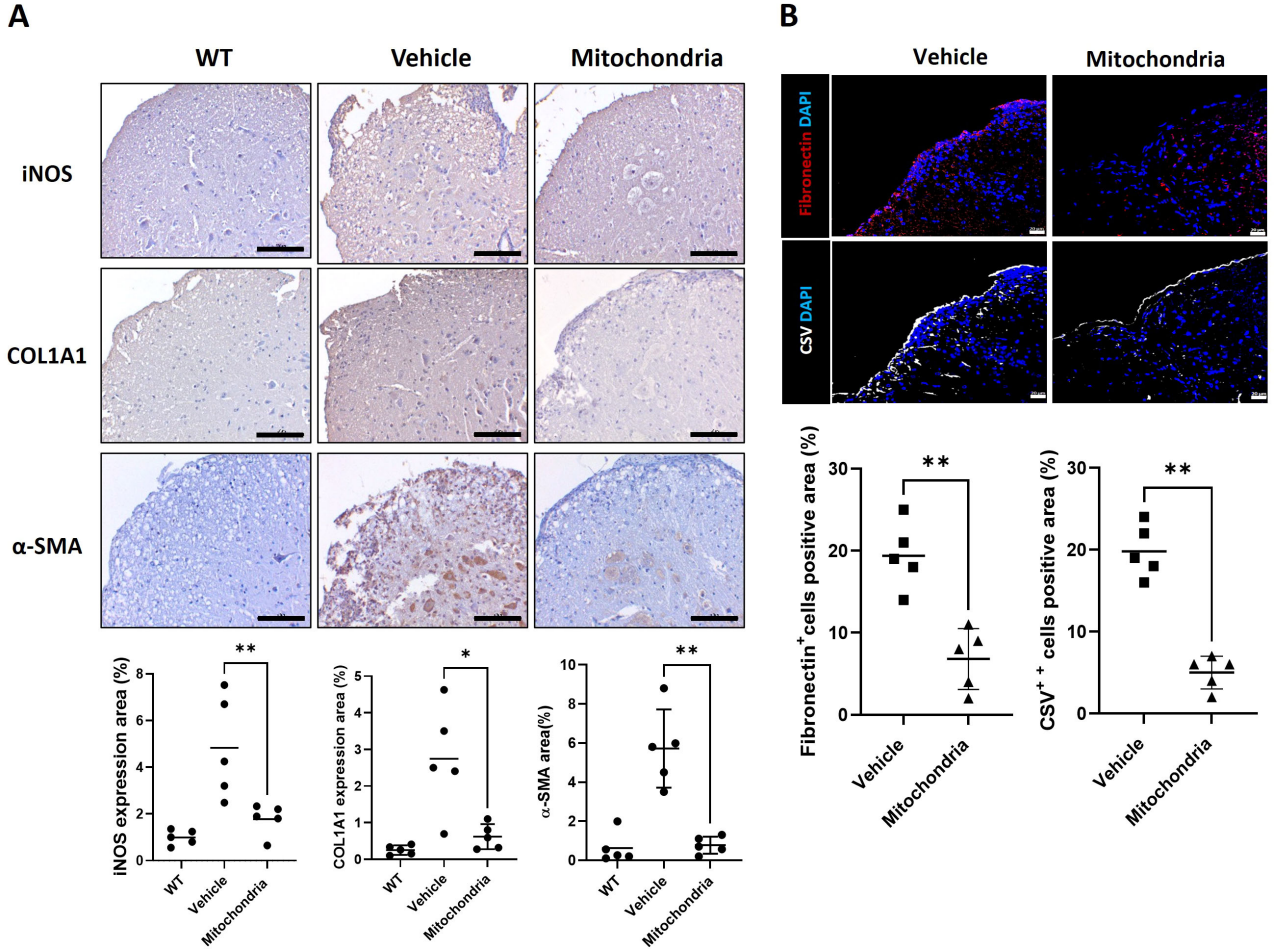
Fibrosis markers after mitochondrial transplantation in spinal cord tissue of an EAE model. **(A)** Representative immunohistochemical (IHC) images showing the expression of iNOS, COL1A1, and α-SMA in spinal cord tissues. Scale bars = 100 μm. **(B)** Confocal immunofluorescence images of Fibronectin (red) and Cell Surface Vimentin (CSV, white) with DAPI (blue) nuclear counterstaining. CSV is used as a marker for activated fibroblasts involved in tissue remodeling. Scale bars = 20 μm. *P < 0.05, **P < 0.01.

To determine whether mitochondrial transplantation influences the formation of a restrictive fibrotic environment that often hinders recovery in chronic neuroinflammation, we examined the expression of markers associated with reactive gliosis and fibrotic scarring in the spinal cord. First, we assessed the expression of inducible nitric oxide synthase (iNOS). While iNOS expression is nearly absent in naïve spinal cord tissue, it serves as a critical mediator of inflammation and nitric oxide (NO) production in EAE lesions, primarily localized in infiltrating macrophages and reactive astrocytes. IHC analysis revealed that the vehicle-treated EAE group exhibited a substantial increase in iNOS-positive areas. However, mitochondrial transplantation significantly reduced iNOS expression (**Figure 4A**), suggesting a suppression of the oxidative and inflammatory milieu mediated by NO. Next, we investigated the impact on fibrotic scarring by measuring key fibrotic factors, including COL1A1, αSMA, fibronectin, and cell surface vimentin (CSV). Specifically, COL1A1 expression was predominantly localized to the meningeal and perivascular fibroblasts, while αSMA labeled reactive astrocytes and micro vessels both indicative of robust glial and fibrotic scar formation. Mitochondrial transplantation resulted in a significant reduction in the positive areas of COL1A1 and αSMA (**Figure 4A**). Immunofluorescence staining demonstrated that the numbers of fibronectin-positive and CSV-positive cells were significantly elevated in the vehicle-treated group, reflecting intense tissue remodeling and scarring. These markers were effectively diminished following mitochondrial transplantation (**Figure 4B**). Taken together, these results demonstrate that mitochondrial therapy effectively inhibits the pathological fibrotic response and glial scarring, potentially facilitating a more permissive microenvironment for tissue repair and axonal stabilization.

### Mitochondrial transplantation alters gut microbiota composition

**Figure 5:**
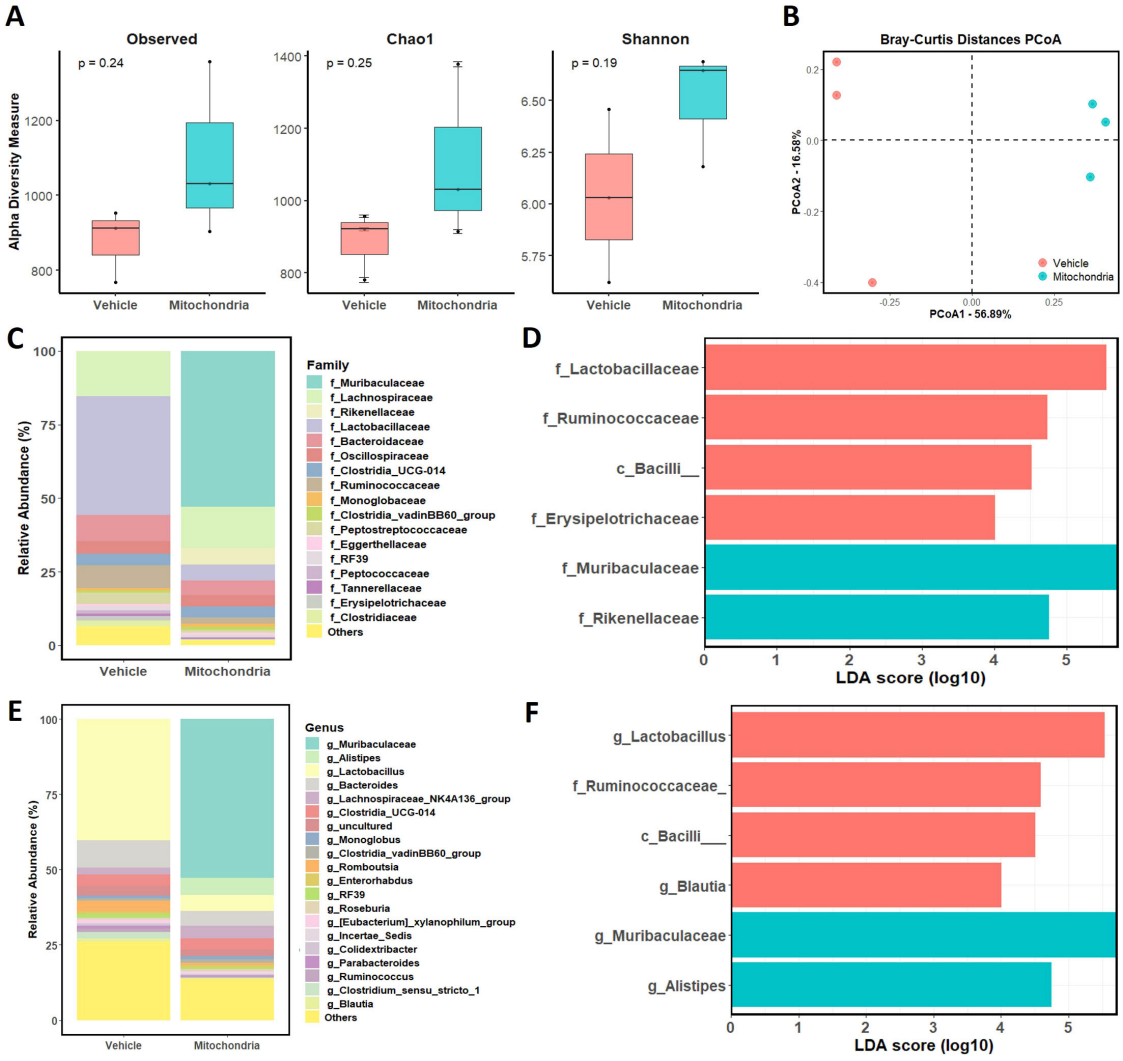
The gut microbiome after mitochondrial transplantation. **(A)** Bar graphs depicting observed operational taxonomic units (OTUs), Chao1 richness estimator, and Shannon diversity index. **(B)** Representative plots showing principal coordinate analysis between vehicle-treated and mitochondrial transplantation mice groups. **(C, D)** Gut microbiome analysis of vehicle-treated and mitochondrial transplantation mice groups at the family level. **(E, F)** Gut microbiome analysis of vehicle-treated and mitochondrial transplantation mice groups at the genus level. Histogram illustrating the linear discriminant analysis scores for bacterial abundances in vehicle-treated and mitochondrial transplantation mice groups.

To investigate whether the systemic administration of mitochondria influences the gut-brain axis, we analyzed the composition and diversity of the gut microbiota in EAE mice. Fecal samples were collected from the vehicle-treated and mitochondrial transplantation groups at the time of sacrifice to evaluate the modulation of the microbial environment. First, we assessed microbial alpha diversity to determine the richness and evenness of the gut community (**Figure 5A**). Although the Observed, Chao1, and Shannon indices showed no statistically significant difference in overall richness (p > 0.05), the mitochondrial transplantation group exhibited a trend toward increased microbial diversity compared to the vehicle group. To further examine the community structure, we performed a Principal Coordinates Analysis (PCoA) based on Bray-Curtis distances (**Figure 5B**). The two groups formed distinct and independent clusters, indicating that mitochondrial transplantation significantly modified the overall gut microbial landscape compared to the vehicle-treated EAE mice. Further taxonomic analysis and linear discriminant analysis effect size (LEfSe) were performed to identify the specific bacterial taxa responsible for these differences (**Figures 5C-F**). A significant increase in the relative abundance of the family *Muribaculaceae* was observed in the mitochondrial transplantation group. This bacterial group is known to be associated with healthy gut function and the production of short-chain fatty acids, which can exert anti-inflammatory effects. Conversely, mitochondrial therapy led to a marked reduction in several taxa that were predominant in the vehicle-treated group, including *Lactobacillaceae* (specifically the genus *Lactobacillus*), *Ruminococcaceae*, and *Bacilli*. Given that these strains have been previously associated with pro-inflammatory environments in EAE models, their reduction suggests that mitochondrial transplantation mitigates gut dysbiosis. Collectively, these results demonstrate that mitochondrial transplantation promotes a shift toward a more homeostatic gut microbiota, potentially contributing to its tissue-protective and immunomodulatory effects via the gut-brain axis.

### Mitochondrial transfer enhances Treg differentiation in MS patient immune cells

**Figure 6:**
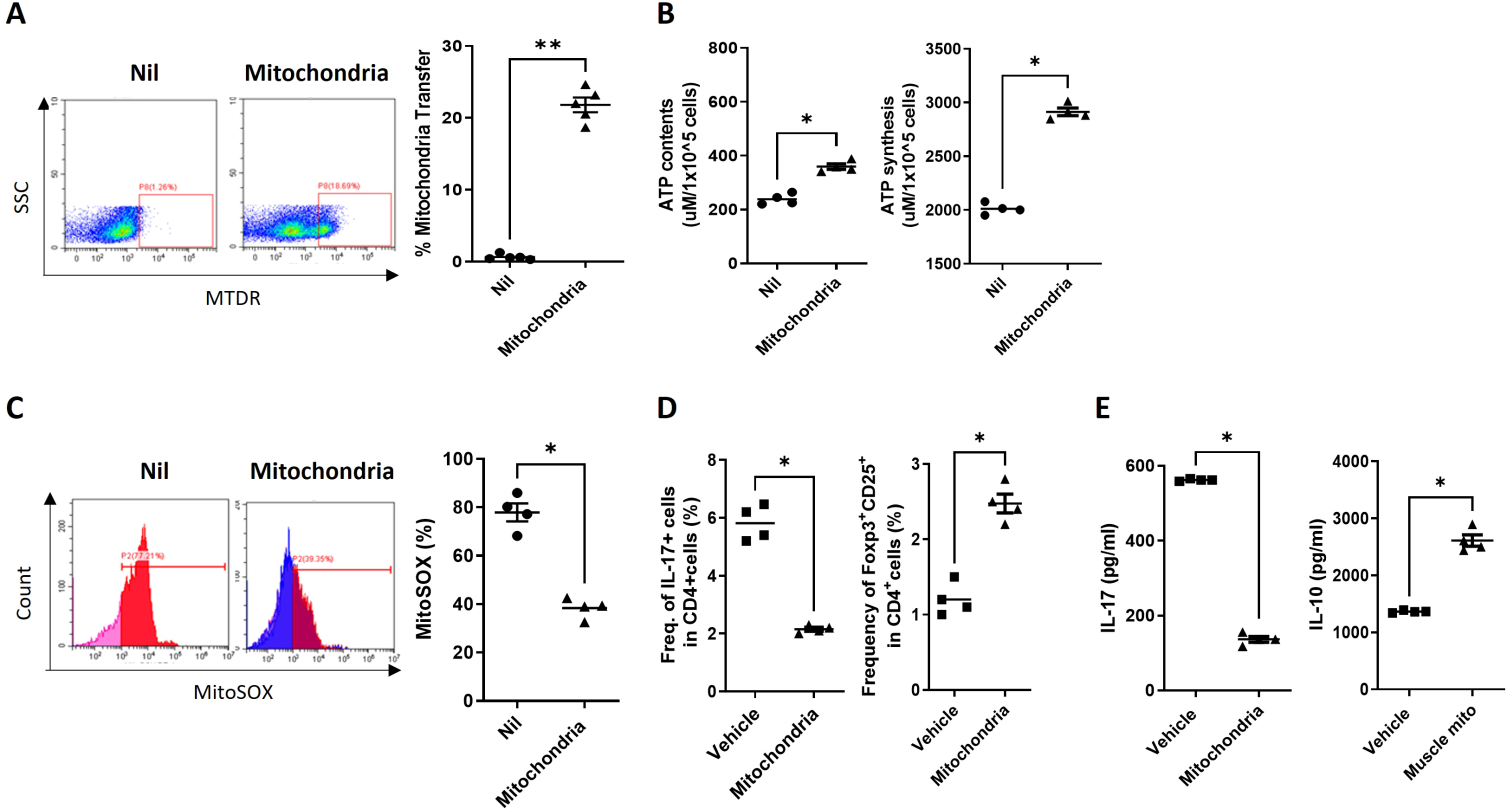
Regulatory T cell differentiation in multiple sclerosis peripheral blood mononuclear cells co-cultured with mitochondria. **(A)** Representative flow cytometry plots showing the internalization of mitochondria in peripheral blood mononuclear cells (PBMCs) from multiple sclerosis (MS) patients cultured in the absence (Nil) or presence (Mitochondria) of isolated mitochondria labeled with MitoTracker Deep Red. **(B)** ATP levels measured in MS PBMCs stimulated with or without mitochondria (5 μg) for 72 hours. **(C)** Mitochondrial reactive oxygen species levels in MS PBMCs assessed by flow cytometry following stimulation with anti-CD3 antibody (0.5 ng/mL) in the presence or absence of mitochondria (5 μg) for 72 hours. **(D)** The frequencies of T helper 17 (Th17) and regulatory T (Treg) cells quantified using flow cytometry after 72 hours of culture with or without mitochondria. **(E)** Concentrations of interleukin-17 (IL-17) and interleukin-10 (IL-10) in culture supernatants collected 72 hours after initiation of co-culture, determined by enzyme-linked immunosorbent assay. *P < 0.05, **P < 0.01. (Nil: untreated control group).

To evaluate the translational potential of our findings from the EAE model, we investigated whether exogenous mitochondrial transplantation could similarly rescue the compromised metabolic and immunologic profiles of human cells. We utilized PBMCs obtained from patients with RRMS. First, to confirm the successful delivery of exogenous mitochondria into the patient cells, we tracked isolated mitochondria pre-labeled with MTDR using flow cytometry. Significant intracellular internalization of the labeled mitochondria was observed in the treated PBMCs compared to the untreated (Nil) group, with the transfer efficiency reaching approximately 20% (**Figure 6A**). This confirms that the exogenous mitochondria are effectively taken up by human immune cells, setting the stage for functional integration. Next, we assessed whether this internalization resulted in a functional metabolic rescue within the MS patient cells. We measured ATP content and synthesis, alongside mtROS production, as critical indicators of bioenergetic health. Following mitochondrial transfer, both ATP content and ATP synthesis were significantly increased compared to the Nil group (**Figure 6B**). Furthermore, the percentage of MitoSOX-positive cells—an indicator of pathological mitochondrial superoxide production—was markedly reduced in the mitochondria-transplanted group (**Figure 6C**). These results demonstrate that the transplanted mitochondria are metabolically active and capable of bypassing the endogenous mitochondrial dysfunction typical of MS pathology. Finally, we examined whether this metabolic recovery could translate into a modulation of the T-cell mediated autoimmune response in humans. Consistent with our EAE results, flow cytometric analysis revealed that mitochondrial transplantation significantly suppressed the frequency of pro-inflammatory Th17 cells while facilitating an increase in Treg differentiation (**Figure 6D**). This cellular shift was further supported by cytokine profiling of the culture medium via ELISA, which showed a significant decrease in the secretion of IL-17 a hallmark effector cytokine of Th17 cells and a concomitant increase in IL-10 a key anti-inflammatory cytokine associated with Treg function (**Figure 6E**). Taken together, these findings provide compelling evidence that mitochondrial transplantation can functionally restore human MS patient immune cells, promoting a switch from a pro-inflammatory to a regulatory state and supporting its potential as a viable clinical intervention.

## Discussion

Our study highlights the potential of mitochondrial transplantation in modulating immune responses, alleviating inflammation, and preserving tissue integrity in an EAE model. The beneficial effects of mitochondrial infusion were evident within 3–5 days and persisted throughout the 22-day observation period. Longer-term durability of this therapeutic effect remains to be elucidated.

In this study, the term ‘mitochondrial transplantation’ refers to the intravenous infusion of isolated mitochondria, a terminology conventionally used in previous studies. While classical allo-MHC rejection is unlikely because mitochondria are not nucleated and do not express surface MHC molecules (25), mitochondrial components such as mtDNA and cardiolipin may activate innate immune sensors. Although no adverse effects were observed in our model, further research is warranted to delineate immune responses to exogenous mitochondria derived from different origins, including autologous and heterologous sources.

Intravenous injection of isolated mitochondria reduced disease severity, which correlated with decreased inflammation and demyelination within the spinal cord. These findings were correlated with reduction of MOG-specific IgG levels and mitochondria-reactive ROS production, suggesting decreased autoimmune response and oxidative stress, respectively. Histological analyses confirmed increase in Treg cells and a decrease in Th17 cells in the splenocyte populations and spinal cord, respectively. Increased expression of mitochondrial markers such as TOM20, PINK1, and TFAM in the spinal cord of mitochondria-treated mice suggests enhanced mitochondrial function and biogenesis within host cells. Although we cannot directly distinguish whether these increases result from internalized exogenous mitochondria or from activation of endogenous mitochondrial biogenesis, the overall findings indicate restoration of mitochondrial homeostasis and cellular energy metabolism following transplantation. Expression of inflammatory cytokines, IL-17 and TNF-α, were also reduced in spinal cord, indicating decreased inflammation in mitochondria-transplanted EAE mice.

In the central nervous system, mitochondria are critical for maintaining axonal transport, synaptic transmission, and neuronal survival by supplying ATP, buffering calcium, and controlling ROS (7, 26). Mitochondrial dysfunction disrupts axonal energy homeostasis and calcium regulation, resulting in axonal degeneration and secondary demyelination, and it also promotes microglial overactivation via oxidative stress and release of mitochondrial danger signals (27). Restoration of mitochondrial function through exogenous mitochondrial supplementation may re-establish local bioenergetic stability, reduce ROS accumulation, and suppress pro-inflammatory microglial phenotypes, thereby attenuating axonal injury (28). These mechanisms may underlie the observed myelin-protective and anti-inflammatory effects of mitochondrial infusion in our EAE model.

Although the exact percentage of mitochondrial uptake was not quantitatively assessed in this study, previous reports have demonstrated that approximately 20–40% of host cells can internalize exogenous mitochondria following intravenous administration (20). Consistent with these findings, we observed a reduction in EAE clinical scores approximately 3–5 days after mitochondrial infusion, suggesting that functional recovery coincided temporally with the expected onset of mitochondrial internalization and bioenergetic restoration.

EAE is an antigen-driven autoimmune model in which immunization against myelin autoantigens triggers robust T cell responses that initiate its pathology with destruction of CNS myelin (29). An altered balance between pro-inflammatory Th17 cells and Treg cells contributes to dysregulated immunity, excessive inflammation, oxidative stress, and demyelination in EAE (30, 31). Therefore, upregulating anti-inflammatory Treg cells, inhibiting pro-inflammatory Th17 cells, and restoring T-cell balance are known as appropriate methods for treating EAE. Mitochondria is known to play a crucial role in regulating the balance between Treg and Th17 cells, which is vital for maintaining immune homeostasis (14). The differentiation and function of these cells are closely linked to cellular metabolic pathways, particularly mitochondrial activity. Th17 cells primarily rely on glycolysis for their energy needs, whereas Tregs depend on OXPHOS and fatty acid oxidation (FAO). The mammalian target of rapamycin (mTOR) pathway promotes glycolysis and supports Th17 differentiation, whereas AMP-activated protein kinase (AMPK) inhibits mTOR, enhancing mitochondrial oxidative metabolism and favoring Treg development. This metabolic distinction influences the Treg/Th17 balance, with mitochondrial function being a key determinant (32, 33). In a former study, metabolic reprogramming of Tregs using peroxisome proliferator-activated receptor (PPAR) agonists ameliorated symptoms in EAE mice, highlighting the therapeutic potential of targeting Treg metabolism (34).

Fibrosis is a frequent pathological reaction to inflammation in many peripheral tissues, and it can inhibit tissue regeneration and repair. It has been reported that remyelination is inhibited by fibrotic responses in MS-related spinal cord injury (35). In this study, administration of mitochondria decreased fibrosis, as well as inflammation and demyelination. Fibrotic markers such as α-SMA and COL1A1 decreased, suggesting that transplanted mitochondria reduced ROS levels by restoring electron transport chain efficiency and scavenging excess ROS. The reduction of ROS protects against mitochondrial DNA damage, maintains cellular homeostasis, and prevents further neurodegeneration, creating a more favorable environment for axonal repair and remyelination. Lowered iNOS levels further supported the anti-inflammatory effects of mitochondrial transplantation. Expression levels of additional fibrosis markers, CSV and fibronectin, in spinal cord tissues were also decreased.

The role of mitochondria in fibrosis of CNS lesions remain incompletely understood, however, previous reports about fibrosis in other organs such as the lungs, liver, and kidneys, provide insights. The fibrosis of those organs is characterized by excessive deposition of EMCs, leading to tissue scarring and impaired function (36). Mitochondrial dysfunction, marked by impaired OXPHOS and increased ROS production, contributes to cellular injury and inflammation, thereby promoting fibrosis. In pulmonary fibrosis, for instance, damaged mitochondria generate excess ROS, leading to cellular stress and apoptosis. Similarly, in hepatic and renal fibrosis, mitochondrial dysfunction exacerbates oxidative stress, furthering fibrotic progression (36). ROS also contribute to fibrosis by stimulating transforming growth factor-beta 1 (TGF-β1), a central factor in fibroblast-to-myofibroblast differentiation and ECM deposition (37). Fibrosis is a hallmark of progressive MS, where excessive scarring in the CNS limits tissue repair (38). Mitochondrial transplantation reduced fibrotic markers such as α-SMA and COL1A1, mitigating ECM buildup and creating a favorable environment for axonal repair and remyelination. These findings align with prior studies showing that fibrosis impedes nerve regeneration and that anti-fibrotic therapies, including pirfenidone and nintedanib, hold potential for MS treatment (38–40).

In our study, the immunomodulatory potential of mitochondrial transplantation was observed *in vitro* as well. In PBMCs co-cultured with isolated mitochondria, increased ATP levels and inhibited mtROS activity was observed. Analysis of T cell activation revealed a suppression of Th17 activity and an increase in Treg differentiation, suggesting improved Treg/Th17 balance. Furthermore, a decrease in IL-17 cytokine levels, associated with Th17 cell activity, and an increase in the anti-inflammatory cytokine IL-10 in the culture medium was observed.

Emerging data reveals that intestinal microorganisms communicate with the mitochondria of mucosal cells, including epithelial and immunological cells. Gut microbiota signaling to mitochondria has been found to modify mitochondrial metabolism, activate immune cells, promote inflammatory signaling, and affect epithelial barrier function (41). Recent studies have indicated that disease progression in MS may result in disruption to intestinal motility, leading to gut microbiota dysbiosis (42). Intriguingly, the present study observed a shift in the microbiome in mitochondria-transplanted EAE mice. The proportion of *Muribaculaceae*, which is known to contribute to gut health and inhibit the growth of pathogenic strains such as *Clostridioides*, increased (43, 44). *Muribaculaceae* and *Alistipes* are also reported to regulate the intestinal environment through the production of short-chain fatty acids (SCFAs). Furthermore, previous studies have demonstrated that *Lactobacillaceae* and *Ruminococcaceae* can contribute to heightened inflammatory responses and compromise the integrity of the intestinal mucosal barrier. Certain members of the *Bacilli* class have been identified as potential pathogens capable of inducing intestinal infections and, in severe cases, progressing to sepsis. In our study, we observed that modifications in gut microbiota composition resulted in a significant reduction in the abundance of these potentially pathogenic bacterial taxa. The observed changes in gut microbiota composition following mitochondrial transplantation suggest a potential interaction between mitochondrial function and the gut microbiome. While the gut microbiome has been the subject of extensive research in the context of neurological disorders, its precise role in MS remains to be elucidated. An analysis of gut microbiota distribution between vehicle and mitochondrial transplantation groups could reveal the presence of distinct microbial communities.

In conclusion, our findings demonstrated that mitochondrial transplantation effectively modulates immune responses, restores mitochondrial dynamics, and reduces inflammation and fibrosis in an EAE model. By addressing both immune imbalance and structural damage, mitochondrial transplantation could represent a promising therapeutic strategy for autoimmune diseases, such as MS. Further understanding the role of mitochondria in MS pathogenesis could offer potential avenues for therapeutic interventions aimed at preserving neuronal integrity and function.

## Data Availability

The raw data supporting the conclusions of this article will be made available by the authors, without undue reservation.
