# Correction: Mitochondrial transplantation ameliorates experimental autoimmune encephalomyelitis by modulating the Th17/Treg balance and restoring metabolic homeostasis

**DOI:** 10.3389/fimmu.2026.1854307

**Published:** 2026-04-27

**Authors:** A Ram Lee, Suh Won Yang, Seon-Yeong Lee, Su Been Jeon, Hye yeon Kang, Jeong Won Choi, Jin Hyung Park, Ju Hyeon Park, Su Bin Son, Yunju Jeong, Jung Hwan Lee, Woojun Kim, Mi-La Cho

**Affiliations:** 1 Lab of Translational ImmunoMedicine, Catholic Research Institute of Medical Science, College of Medicine, College of Medicine, The Catholic University of Korea, Seoul, Republic of Korea; 2 Department of Pathology, College of Medicine, The Catholic University of Korea, Seoul, Republic of Korea; 3 Mitochondrial Immunomedicine center for Global Health Therapeutics (MIGHT), Catholic Research Institute of Medical Science, College of Medicine, The Catholic University of Korea, Seoul, Republic of Korea; 4 Department of Biomedicine and Health Sciences, College of Medicine, The Catholic University of Korea, Seoul, Republic of Korea; 5 Department of Food and Nutrition, College of Human Ecology, Kyung Hee University, Seoul, Republic of Korea; 6 Department of Neurology, Seoul StMary’s Hospital, College of Medicine, The Catholic University of Korea, Seoul, Republic of Korea

**Keywords:** experimental autoimmune encephalomyelitis (EAE), mitochondria, multiple sclerosis, spinal cord, T cell

In the published article, there was an error in the **Funding** statement. The **Funding** statement was displayed as:

“The author(s) declared that financial support was received for this work and/or its publication. This work was supported by the National Research Foundation of Korea (NRF) grant funded by the Korea government (MSIT) (RS-2025-23323617), GRDC Cooperative Hub (Grant number RS-2023-00259341) through the National Research Foundation of Korea funded by the Ministry of Science and ICT and National Research Foundation of Korea (NRF) funded by the Ministry of Education (RS-2024-00449728).”

The correct **Funding** statement is:

“This work was supported by the National Research Foundation of Korea (NRF) grant funded by the Korea government (MSIT) (RS-2025-23323617, RS-2024-00454685), GRDC Cooperative Hub (Grant number RS-2023-00259341) through the National Research Foundation of Korea funded by the Ministry of Science and ICT and National Research Foundation of Korea (NRF) funded by the Ministry of Education (RS-2024-00449728).”

The original version of this article has been updated.

